# Auxin sensing is a property of an unstructured domain in the Auxin Response Factor ETTIN of *Arabidopsis thaliana*

**DOI:** 10.1038/s41598-018-31634-9

**Published:** 2018-09-10

**Authors:** Sara Simonini, Philippe J. Mas, Caroline M. V. S. Mas, Lars Østergaard, Darren J. Hart

**Affiliations:** 1 Crop Genetics Department, John Innes Centre, Norwich Research Park, Colney Lane, NR4 7UH Norwich, UK; 2 Integrated Structural Biology Grenoble (ISBG) CNRS, CEA, Université Grenoble Alpes, EMBL, 71 avenue des Martyrs, F-38042 Grenoble, France; 3 Institut de Biologie Structurale, CEA, CNRS, Université Grenoble Alpes, 71 avenue des Martyrs, F-38042 Grenoble, France; 4 Present Address: Department of Plant and Microbial Biology, University of Zürich, Zollikerstrasse 107, CH-8008 Zürich, Switzerland

## Abstract

The plant hormone auxin regulates numerous aspects of the plant life cycle. Auxin signalling is mediated by auxin response factors (ARFs) that dimerise with modulating Aux/IAA repressors. ARF3 (ETTIN or ETT) is atypical as it does not interact with Aux/IAA repressors. It is proposed to be a non-canonical auxin sensor, regulating diverse functions essential for development. This sensing ability relies on a unique C-terminal ETT specific domain (ES domain). Alignments of ETT orthologues across the angiosperm phylum revealed that the length and sequence identities of ES domains are poorly conserved. Computational predictors suggested the ES domains to be intrinsically disordered, explaining their tolerance of insertions, deletions and mutations during evolution. Nevertheless, five highly conserved short linear motifs were identified suggesting functional significance. High-throughput library screening identified an almost full-length soluble ES domain that did not bind auxin directly, but exhibited a dose-dependent response in a yeast two-hybrid system against the Arabidopsis INDEHISCENT (IND) transcription factor. Circular dichroism confirmed the domain was disordered. The identification and purification of this domain opens the way to the future characterisation of the ETT auxin-sensing mechanism *in planta* and an improved understanding of auxin-mediated regulation.

## Introduction

The plant hormone auxin (indole-3-acetic acid; IAA) is involved in almost every aspect of the plant life cycle and guides crucial developmental decisions during organ growth and differentiation^1,2^. Canonical nuclear auxin signalling, which provides interpretation of auxin cellular concentration and translation of the latter into a precise transcriptional outcome, relies on the activity of the auxin response factors (ARFs), which in turn are regulated by the Aux/IAA repressors^3^. At low cellular concentrations of auxin, the ARFs are kept in an inactive state through heterodimerisation with the Aux/IAA repressors. When auxin cellular concentrations increase, the Aux/IAA repressors are ubiquitinated and degraded, thus releasing the ARFs to regulate transcription of their target genes.

The Arabidopsis genome encodes 23 ARFs with a conserved domain organisation: a DNA binding domain (DBD), located at the N terminus^4^, and the PB1 (Phox and Bem1) domain at the C terminus that mediates binding to the Aux/IAA repressor and other ARF members^4,5^. Crystal structures of the highly conserved ARF1 and ARF5 DBDs of *A. thaliana* have been determined, the former in complex with its auxin response DNA element. The DBDs were observed to homodimerise with specificity of different ARFs conferred by half-site spacing^4^.

**Figure 1:**
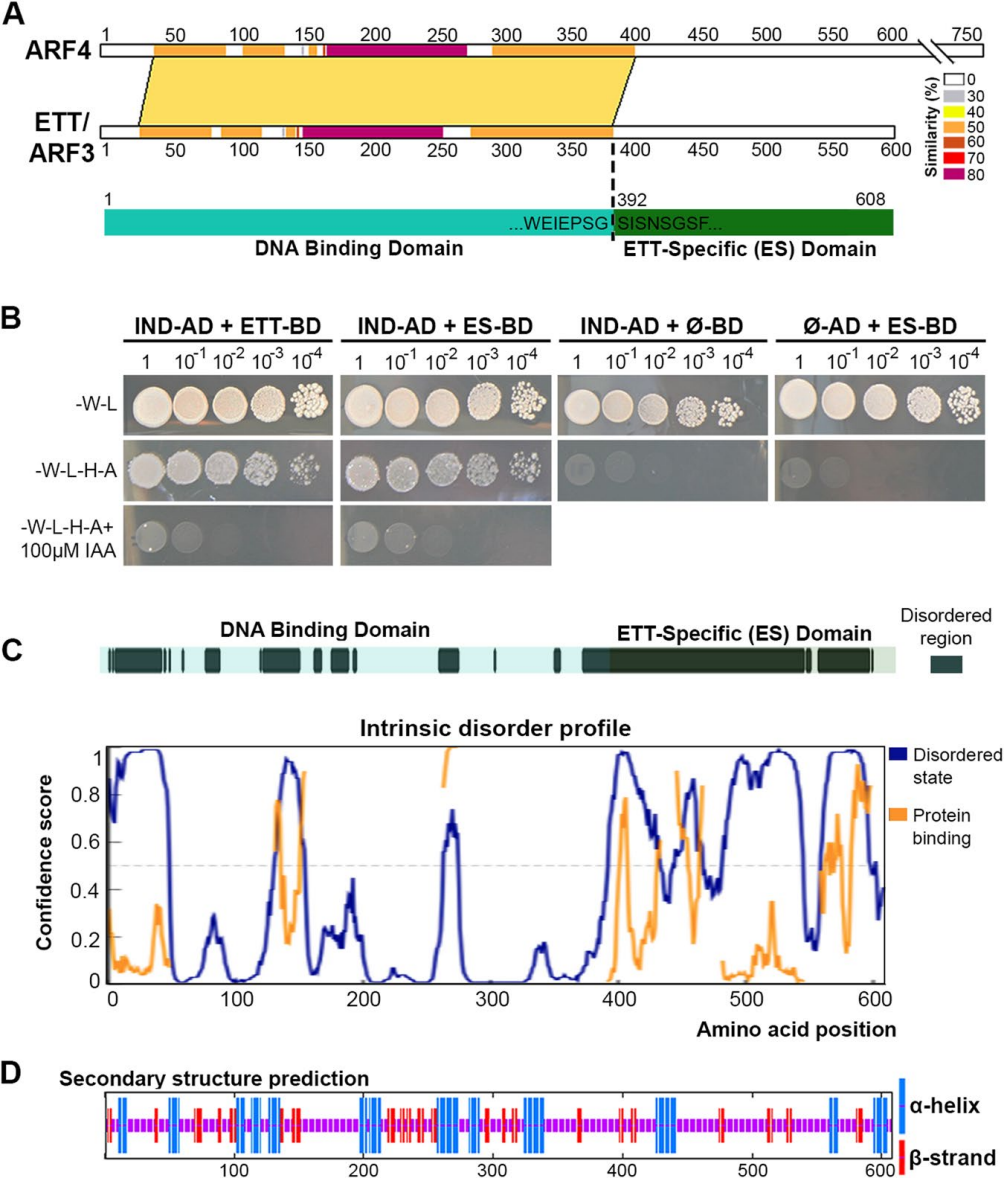
Analyses of ETT domains, order-disorder prediction and secondary structure prediction. (**A**) Protein sequence alignment by SIM between ETT/ARF3 and its closest homologue ARF4. Different colours indicate percentage similarity. Below, schematic representation of the ETT showing limit of DNA binding domain (cyan) as determined by alignment with ARF4 and the following ETT-Specific (ES) domain (dark green). (**B**) Y2H assay between the ETT and IND, an *A. thaliana* transcription factor previously identified as ETT interactor^8^, and ETT-ES domain and IND. Interaction is observed on the selective plate -W-L-H-A. Yeast growth is inhibited by IAA in the media (lower panel). (**C**) A disorder prediction and putative protein binding regions for full-length ETT by PrDos and DISOPRED3 servers. (**D**) Secondary structure prediction by SOPMA.

Three atypical ARFs: ARF3, ARF13 and ARF17, exhibit a different domain arrangement since they lack the PB1 domain^5,6^. This suggests they do not interact with Aux/IAA repressors^7^ and may thus function differently from canonical auxin signalling. However, in contrast to ARF13 and ARF17 that are simply truncated ARFs^6^, ARF3, also known as ETTIN (ETT), possesses a C-terminal domain that is not observed in other eukaryotic or prokaryotic proteins^8^. An alignment of ETT and its closest homologue ARF4 reveals the canonical ARF family DBD (residues 1–391) and this ETT-specific domain (ES domain; residues 392–608) (Fig. 1A).

The ES domain has been proposed recently to facilitate the ETT-mediated response to auxin cellular concentration^8^. The molecular mechanism remains to be unravelled; however, several independent methods have revealed that interaction between ETT and a range of transcription factors is affected by the presence of auxin. The interaction between ETT and INDEHISCENT (IND) is inhibited by the addition of IAA in a yeast-two-hybrid (Y2H) assay^8^. Interestingly, this effect was specific to the naturally occurring IAA molecule, since synthetic auxin analogues NAA (1-naphthaleneacetic acid) and 2,4D (2,4-dichlorophenoxyacetic acid) did not affect ETT heterodimerisation by Y2H^8^. In *A. thaliana*, mutational disruption of the ES domain induces developmental aberrations in different plant organs (lateral roots, branches, ovules and carpel) suggesting that ETT-mediated auxin-sensing is essential for correct organ morphogenesis^8^.

The direct effect of auxin on ETT heterodimerisation observed in Y2H assays suggested that ETT may interact with the IAA molecule, perhaps in a similar manner to ligand-binding by nuclear steroid hormone receptors in animals^9^. In order to investigate this hypothesis, we sought to carry out a detailed bioinformatics and biophysical characterisation of purified recombinant ETT protein combined with Y2H assay. The results revealed that the ES domain is predominantly intrinsically unstructured, but bears striking motifs that are almost universally conserved, both in sequence and in linear organisation, across evolutionarily diverse ETT homologues. The identification of these motifs allowed us to interpret *A. thaliana* phenotypic studies on ETT deletion and point mutants. A 27 kDa engineered form of the ES domain was identified by the high throughput ESPRIT method^10,11^ that could be produced and purified at multi-milligram quantities, and yet exhibited identical auxin responses to the wild-type sequence in Y2H assay. This opens the way for structural and biophysical studies into mechanisms of non-canonical auxin-mediated plant responses following identification of the molecular partners that interact with the conserved linear motifs in the ES domain.

## Results

### Bioinformatic analysis of domain composition in ETT

**Figure 2:**
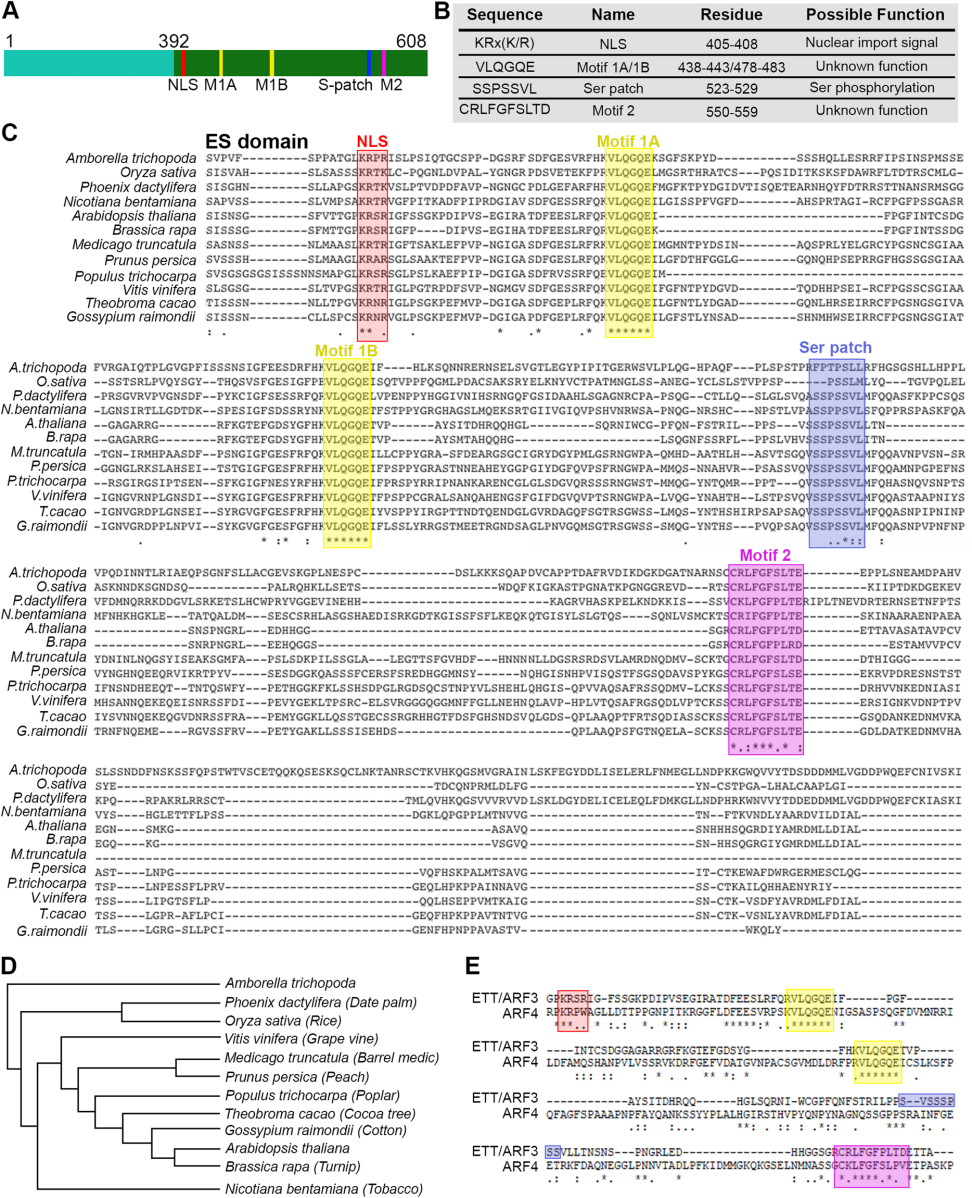
Four motifs are conserved in the ES domain across angiosperms. (**A**,**B**) Schematic representation of the conserved motifs identified in the ES domain (**A**) and their sequence and possible function (**B**). (**C**,**D**) Alignment of ES domain from different angiosperm species (**C**) and their phylogenetic tree (**D**). Motif colour code: red, NLS; yellow, Motif 1A and 1B; blue, Ser patch; purple, Motif 2. (**E**) Alignment between ETT/ARF3 and ARF4 highlighting conservation of NLS, Motif 1A and 1B and Motif 2 between the two proteins.

Analysis of ETT (UniProt O23661) using the Basic Local Alignment Search Tool (BLAST) revealed a clear sequence conservation in the N-terminal DBD region across ARF family members, but not in the C-terminal ES domain (Fig. 1A). The ES domain is both necessary and sufficient to mediate the IAA-sensitive interaction with the protein partner IND (Fig. 1B). Predictors of disorder were used to assess the foldedness of the ES domain. PrDOS^12^, DISOPRED3^13^, IUPred^14^ all predicted high levels of disorder, in contrast to the upstream DBD (Fig. 1C and Supp. Fig. 1A). DISOPRED3 and IUPred further predicted short regions within the ES domain with characteristics found in protein-interacting motifs (Fig. 1C and Suppl. Fig. 1A). A secondary structure analysis using PSIPRED^15^ predicted only two short helices in the whole ES domain (residues 392–608) (Supp. Fig. 1B) with 196/217 (90%) of residues annotated as random coil. Similarly, another secondary structure predictor, SOPMA^16^ predicted low secondary structure content (Fig. 1D). The Phyre2^17^ server attempts to predict structural similarities in distantly related proteins; here it identified no such similarities with the ES domain, predicting 76% of this region to be disordered (Supp. Fig. 1C). Phyre2 generates an alignment of distantly related sequences identified via PSI-BLAST^18^ that revealed five short highly conserved regions within the ETT C-terminal domain that otherwise varied highly in length and sequence identity (Fig. 2A–C). We used this PSI-BLAST output to guide the manual alignment of ETT members from a diverse evolutionary collection of angiosperm species, both mono- and dicotyledon (Fig. 2C,D). A broader comparison of annotated ETT protein sequences reveals these regions to be almost completely conserved in otherwise highly variant sequences including *Amborella tricopoda* (Fig. 2C,D), the only living species of a sister clade to extant flowering plants^19^.

We attempted to predict the functions of these regions using the Eukaryotic Linear Motif (ELM) database^20^ which identified the KRx(K/R) motif as a classic mono-partite nuclear localisation signal (NLS) and the SSPSSVL motif, that we called the Ser patch, as the site of phosphorylation by multiple kinases (Fig. 2B). The tandem repeated VLQGQE motif, called Motif 1A and 1B, and the downstream CRLFGFSLTE motif (Motif 2) were not identified by ELM and we could not find functional descriptions of these motifs elsewhere. Across the twenty-three Arabidopsis ARF family members, the predicted NLS is found in a similar position in 14 ARFs (Supp. Fig. 2). The closest ARF family member to ETT is ARF4: this contains a shorter version of the Motif 2 and the tandem repeated Motif 1A and Motif 1B (Fig. 2E) but lacks the full NLS motif and Ser patch. The identification of highly conserved motifs across ETT homologues in evolutionary distant plant species suggests that the auxin-sensing ability of ETT is of ancient origin.

### Strategy for random truncation libraries of ETT

**Figure 3:**
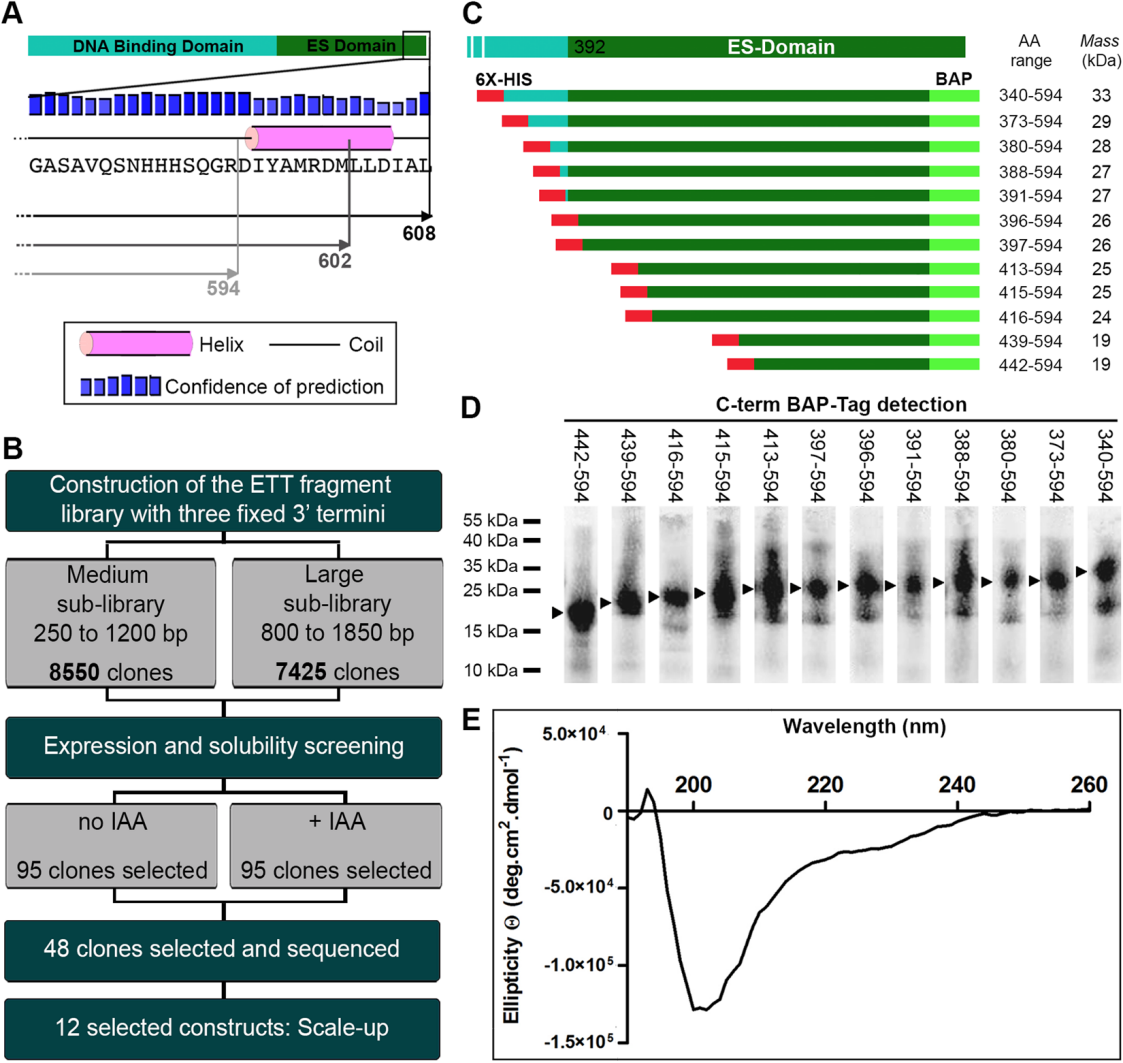
Screening for expression and solubility from a ETT random truncation library. (**A**) Schematic representation of the three ETT C termini used in N-terminal truncation experiments: aa 1–608, 1–602 and 1–594. (**B**) Schematic representation of the strategy for identifying the soluble ES domain of ETT. From a high throughput screen of 15,975 clones, 12 clones were selected for 1 litre scale-up. (**C**) Summary of sequenced ETT constructs yielding purifiable fragments after 4 mL scale-up. Construct boundaries are shown with the predicted molecular weights including 5 kDa from the hexahistidine and biotin tags. (**D**) Fluorescent streptavidin blot against the C-terminal BAP for protein fragments summarised in (**C**). Uncropped gel shown in Supp. Fig. 4A,B. (**E**) CD analysis of the ETT_388-594 construct showing characteristic spectrum of an unstructured protein.

Although the approximate region of the ETT domains seems clear, precise definition of well-behaving soluble ETT constructs, especially of the ES domain, is complicated by the lack of similar proteins for sequence alignments. Our initial attempts to sub-clone constructs yielded only insoluble material, while the full-length protein was poorly expressed, insoluble and toxic. We therefore applied the ESPRIT (expression of soluble proteins by random incremental truncation) method^10,11^ to identify a soluble and well-expressing fragment of ETT containing most or all of the ES domain. Three different fixed C termini were defined as possible end points of ETT against which the N terminus would be randomly truncated. These comprised the native C terminus (residue 608) and two short deletions terminating at residues 602 and 594 that truncated a predicted 11 residue α-helix (Fig. 3A). DNA fragments encoding ETT 1–608, 1–602 and 1–594 were subcloned into pESPRIT002^11^, a reduced-size pET-derivative, downstream of an N-terminal TEV protease-cleavable hexahistidine tag and upstream and in-frame with a C-terminal biotin acceptor peptide (BAP) used as a marker of protein solubility and stability^10,21^. The three sequence-verified plasmid constructs were pooled at equimolar concentrations, then plasmids linearised and truncated with exonuclease III and mung bean nuclease as described^11^. Two sub-libraries were generated based on their size range: a medium sub-library (250–1200 bp) that spanned the suspected ES domain, and a large sub-library (800–1850 bp) that additionally included the region encoding the DBD (Fig. 3B). Following transformation of a highly competent cloning strain (MACH1), colony PCR of 34 randomly selected clones from each sub-library revealed an even distribution of construct sizes (Supp. Fig. 3). Sub-library plasmids were then used to transform *E. coli* BL21 AI RIL for protein expression analysis, and a total of 15975 colonies (8550 for the medium sub-library and 7425 for the large sub-library) were picked into 384-well plates, comprising an approximate three-fold oversampling of constructs.

### High throughput expression and purification screening for soluble constructs

Clones from each sub-library were robotically arrayed as inocula from 384-well plates onto nitrocellulose membranes laid over LB agar trays. Protein expression in growing colonies was induced by placing the membranes on agar trays containing L-arabinose and biotin only or L-arabinose, biotin and IAA, the latter to see whether IAA enhanced protein expression through ligand-induced stabilisation. Clones expressing putatively soluble constructs were identified by hybridising the membranes simultaneously with Alexa488-streptavidin against the biotinylated C-terminal tag, and with a monoclonal anti-hexahistidine tag antibody with associated Alexa532 mouse secondary antibody, against the N-terminal hexahistidine tag. No obvious difference was observed when comparing signals in the presence and absence of IAA. Pixel intensity values from C-terminal biotin and N-terminal hexahistidine tags were extracted for each arrayed colony and imported into a spreadsheet for analysis. Clones with no detectable hexahistidine signal were discarded and remaining clones were ranked according to their biotin intensity. Data from colony filters screened both with and without IAA was treated separately and the highest ranking 95 clones, almost all from the medium sub-library, were isolated from the library (Fig. 3B). The 95 selected ETT clones from IAA-minus and IAA-plus screens were expressed in 4 mL liquid cultures in 24-well plates along with a positive control expressing maltose-binding protein. Cells were lysed and the hexahistidine-tagged proteins purified with Ni^2+^-NTA agarose beads in a 96-well filter plate. Imidazole elution fractions were analysed by fluorescent streptavidin blot after SDS-PAGE and transfer to nitrocellulose membranes (Supp. Fig. 4A,B). Based on the quantity and level of proteolysis of purified proteins, 48 clones (27 from IAA-minus and 21 from IAA_plus screens) exhibiting sizes of 15–35 kDa were isolated, plasmids extracted and sequenced. Interestingly, all 48 selected clones harboured the truncated aa 594 C terminus, indicating that the deletion of the small C-terminal helix was necessary to confer soluble expression of the ETT ES-domain in *E. coli*. Colony PCR of randomly selected clones prior to screening steps confirmed the presence of all three fixed C-termini in the library (Supp. Fig. 4C).

### Characterisation of constructs

Twelve constructs spanning 19–28 kDa (Fig. 3C) were selected for larger scale expression trials. Streptavidin blot of Ni^2+^-NTA small-scale purified constructs showed a continuum of sizes, consistent with a lack of compact globular structure (Fig. 3D). Based upon these preliminary results, 3 L cultures were performed for selected clones: ETT_439-594 of 21.6 kDa, ETT_388-594 of 27.6 kDa and ETT_373-594 of 28.8 kDa. All three constructs yielded approximately 3 mg of protein after Ni^2+^-NTA affinity purification. The longest protein, ETT_388-594, contained the entire predicted ES domain except for the C-terminal α-helix, and was selected for subsequent studies following further optimisation of the purification protocol (see methods and Supp. Fig. 5A).

**Figure 4:**
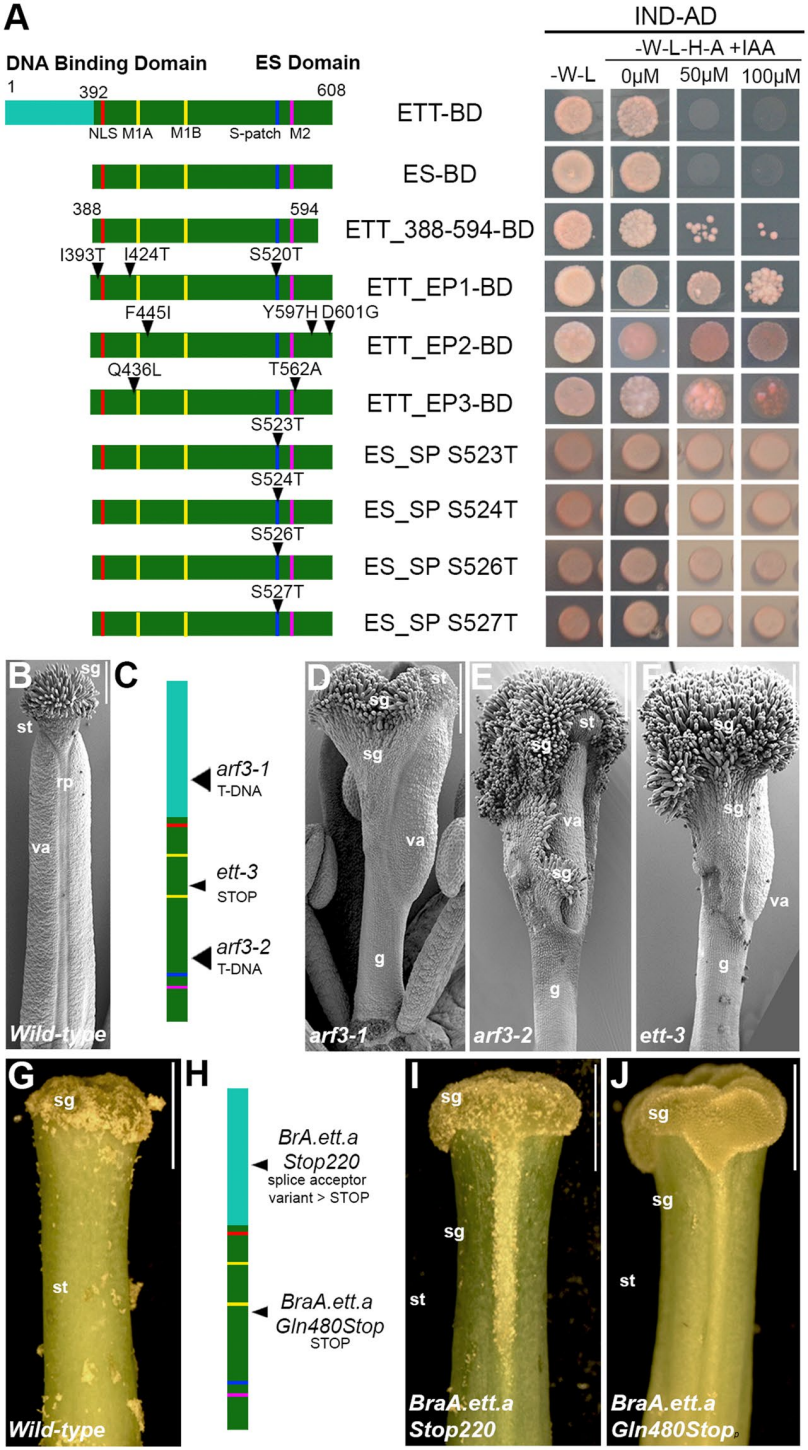
ETT 388–594 is the auxin-responsive domain. (**A**) Schematic representation of ETT variants and their behaviour in Y2H assay in combination with IND and in the absence and presence of auxin at different concentrations. Clones EP1, 2 and 3 were isolated from a random mutagenesis analysis of the ES domain; clones called ES_SP, referring to the Ser patch, are serine to threonine mutations introduced by site-specific mutagenesis. (**B**–**E**) Mapping and Scanning electron microscopy images of gynoecium of annotated *A. thaliana* wild-type (**B**) and *ett* mutant: *arf3-1* (**D**), *arf3-2* (**E**) and *ett-3* (**F**). (**G**–**J**) Mapping and optical microscopy images of gynoecium of *Brassica rapa* wild type (**G**) and *ett* mutant: *BraA.ett.a Stop220* (**I**) and *BraA.ett.a Gln480Stop* (**J**). Abbreviations: rp, replum; sg, stigma; st, style, va, valves. Scale bars: 100 μm (**B**,**D**,**E**), 1 mm (**G**,**I**,**J**).

Analyses by Y2H revealed the ETT_388-594 construct retained full responsiveness to increases in auxin concentration (Fig. 4A; ETT-BD and ES-BD and ETT-388-594-BD rows) confirming it to be a stable and purifiable form of the ETT auxin-responsive domain capable of binding the Arabidopsis INDEHISCENT (IND) transcription factor used as the bait in the assay. To probe the auxin sensing ability of the ES domain, random mutations were introduced into the ES domain sequence by error prone mutagenesis. A pool of ES mutants was cloned and tested in Y2H assay in the presence of IAA to isolate viable clones exhibiting IAA insensitivity. Three ES domain variants were isolated (Fig. 4A, clones ES_EP1-3 BD) harbouring amino acid substitutions at the beginning (Fig. 4A, I393T, I424T, F445I) and the end (Fig. 4A, T562A, Y597H, D601G) of the ES domain. Mutations were also identified in proximity to the Motif 1B (Fig. 4A, Q436L) and in the Ser patch (Fig. 4A, S520T) which impair the auxin sensing property of the ETT-IND dimer. In parallel to the error-prone approach, we also introduced manually point mutations into the Ser patch by site-specific mutagenesis (S523T, S S524T, S526T and S527T; Fig. 4a). Interestingly, these specific mutations within the Ser patch strongly inhibited auxin perception.

In order to characterise the ES domain in purified form, an expression and purification protocol was developed yielding soluble protein at the multi-milligram levels required for structural and biophysical techniques. Six milligrams of purified protein was obtained from 2 L of bacterial culture with limiting amounts of Ni^2+^-NTA agarose. Elution fraction 10 comprised a strong major species and a minor component of lower molecular weight (Supp. Fig. 5A). Analysis by LC ESI-TOF mass spectrometry revealed the major species (27,192 Da) had the expected weight of ETT_388-594 (including hexahistidine and biotin peptide tags), minus the N-terminal methionine. The minor lower molecular weight species shifted in size upon TEV digestion (Supp. Fig. 5B), identifying it as a truncated form of the ES domain; the mass (18,014 Da) corresponded closely to hexahistidine-tagged ES-388-530 minus the N-terminal methionine. Analysis of protein in fraction 10 by circular dichroism (CD) revealed only unstructured polypeptide (Fig. 3E) in agreement with the bioinformatic predictions. Furthermore, the purified ES domain was unaffected by incubation at 95 °C for 1 h (Supp. Fig. 5B), a well-known characteristic of some intrinsically disordered proteins (IDPs)^22^. The clear absence of structure, and therefore potential for an auxin binding pocket, explained the failure to detect auxin binding by ITC in preliminary experiments (not shown).

### Biological function of the ES domain

The female reproductive organ, the gynoecium, develops at the centre of the flower and it is composed of several different tissues with specific function (Fig. 4B,G): the style and stigma at the top, fundamental for pollen recognition and germination; two elongated valves that protect and enclose the developing ovules; and the replum, which separates the two valves and offers mechanical support. This precise tissue and organ sub-division is completely lost in *A. thaliana ett* mutants that harbour premature translational interruption in the DNA binding domain and thus delete the region identified in this study (Fig. 4C,D). Indeed, gynoecia from the *arf3-1* mutant allele (*A. thaliana*)^23^ (Fig. 4D) develop with reduced valve length and width, an absence of replum and conspicuous growth of stigmatic tissue. Consistent with a role for ETT in the specification of tissue identity and patterning in female reproductive structures, ectopic growth of stigmatic tissue and irregularities at the gynoecium top can be observed along the style of the *Bra.A.ett.a Stop220*^23^ mutant (*Brassica rapa*, accession R-o-18) (Fig. 4H,I). Similar phenotypes are also observed in *ett* mutants that harbour a premature stop codon or T-DNA insertion in the ES domain. For example, gynoecia from *arf3-2*^23^, *ett-3*^8,24^ (Fig. 4E,F) and *Bra.A.ett.a-Gln480Stop* mutants (Fig. 4J) typically show severe and extreme phenotypes such as poor tissue identity and definition and ectopic growth of stigmatic tissue. This genetic evidence supports a fundamental role in the biological and functional contribution of the ES domain and motifs therein to the complete spectrum of ETT activities.

## Discussion

The Auxin Response Factor ETT (ARF3) of *A. thaliana* is essential for correct female reproductive structure development^8,25^. For decades ETT has been proposed to act as an interpreter of auxin concentration in the female reproductive organ, but only recently has a molecular mechanism been proposed whereby auxin affects the interaction between ETT and protein partners, including transcription factors, leading to changes in the expression of downstream targets^8,25^. Such a mechanism may involve ETT binding directly or indirectly to auxin in a concentration-dependent manner. To study this interaction *in vitro* using purified components, we previously attempted to produce recombinant full-length ETT but encountered problems of low yield, insolubility and toxicity to host organisms. Similar problems occurred when attempting to express the ES domain alone using *E. coli*, yeast, insect cells and cell-free expression systems. One complicating factor in expressing the ES domain is that design of expression constructs proved difficult since BLAST yielded no similar sequences, thus sequence alignments could not be assembled for domain identification. To overcome this problem, we used ESPRIT, a random-based library construct screening process, to identify regions of ETT that could be over-expressed in *E. coli*. The usefulness of this method for empirical dissection of intractable proteins into manageable folded domains has been demonstrated for a diverse collection of proteins^11,26^ including the *A. thaliana* SEP3 transcription factor^27^. During the course of the ESPRIT screen, we performed a deeper bioinformatic appraisal of the ES domain of ETT. Our analysis using a range of structural prediction servers indicated that ETT belongs to the class of intrinsically disordered proteins (IDPs), with approximately 90% of its residues present as random coil i.e. lacking significant secondary and tertiary structure. This was subsequently confirmed by CD spectroscopic and thermal stability analyses on purified ES domain. Despite their lack of structure, IDPs are often functional and have diverse roles in forming protein-protein interactions in processes such as signalling and gene regulation^28–30^. A common characteristic of IDPs is the presence of short linear motifs (SLiMs) of typically fewer than 10 amino acids that form stable or transient complexes with other biomolecules^31^. Analysis of functionally equivalent ETT proteins from many plant species revealed that the length and sequence identities of their ES domains were poorly conserved, consistent with the prediction that these regions possess no folded domains and therefore tolerate sequence insertions, deletions and mutations during evolution. However, four SLiMs were identified as being almost invariably present and always in the same linear order (Fig. 2).

ESPRIT has been used previously on IDPs to express long unstructured polypeptides (100–200 residues) for biophysical and NMR studies^32,33^. Thus, although the experiment here was originally conceived to identify folded ES domain constructs, the results obtained are consistent with the predictions of intrinsic disorder, notably that a continuum of construct lengths were obtained, all being soluble and purifiable (Fig. 3C,D and Suppl. Fig. 4A,B). The design of the experiment, in which all 5′ unidirectional truncations of the *ETT* coding region were generated against three fixed 3′ ends, revealed that only those lacking a predicted 11 residue C-terminal α-helix produced protein, suggesting that deletion of this helix is necessary to obtain the ETT ES domain in *E. coli* at levels above the threshold of the high-throughput screen. Combining the results of the screen with the informatic predictions, a 27 kDa ETT variant ETT_388-594, encompassing the entire predicted ES domain (except the short C-terminal truncation) and including the four identified SLiMs, was selected for further functional studies.

Circular dichroism and thermal stability analyses confirmed the bioinformatic predictions of near-complete disorder of the ES domain, explaining earlier failed efforts to detect direct binding of auxin and analogues to purified ETT_388-594 *in vitro* using ITC (data not shown). This may also suggest why auxin presence during the ESPRIT screen or scale-up expression elicited no difference in construct behaviour (Supp. Fig. 4A,B). However, once cloned into the Y2H system, the ETT_388-594 construct exhibited full responsiveness to exogenous auxin (Fig. 4A). Thus, auxin sensing is an inherent property of the ETT_388-594 fragment in this system, and presumably *in planta*. The inability to detect direct auxin binding to purified ETT *in vitro* may be expected since an IDP will not contain a classical ligand binding pocket in isolation. By contrast, the auxin-sensing protein complex constituted in the Y2H assay requires heterodimerisation with transcription factors (e.g. IND, Fig. 1B) to elicit a response. Based on our results, we suggest a model of auxin-sensing in which ETT heterodimerises with partners, including Arabidopsis transcription factors belonging to bHLH, HOMEOBOX, AP2-Domain and YABBY families, to generate an auxin-sensing conformation. The molecular-level details remain unclear, but we speculate that it might involve the formation of an auxin-binding pocket, either in the heterodimer interfacial region, or via an allosteric mechanism whereby an auxin-binding conformation in one partner is induced upon binding the other.

Regarding the functions of the SLiMs, the prediction of a NLS by ELM is unsurprising due to the nuclear localisation of the ETT transcription factor^8^. The Ser patch is predicted to be a phosphorylation site of multiple kinases. A functional significance of this motif is suggested by specific and random mutagenesis results where substitution of serine to threonine induced auxin insensitivity^8^. The functions of the highly conserved Motif 1A, 1B and Motif 2 remain unclear, but clues about their function may be provided from Arabidopsis and *Brassica rapa ett* mutants with partial truncations of the ES domain that exclude the Ser patch and Motif 2; these result in phenotypic changes during gynoecium development (Fig. 4B–J). Moreover, auxin-insensitive ETT variants can be obtained by introducing specific point mutations in the ES domain^8^ (Fig. 4A). Therefore, the newly identified ETT 388_594 domain appears to be the same entity that mediates the effect of IAA on ETT-protein interactions. It seems possible that some of the SLiMs are direct docking sites for plant transcription factors, but further investigation will be required involving interaction screens to identify partners, mutational analyses *in vitro* and phenotypic studies *in planta*.

In this study, we have advanced the knowledge on the auxin sensitivity mechanism by delineating a functional region of ETTIN (ETT_388-594) that exhibits full sensitivity in Y2H. It can be over-expressed and purified, providing a useful tool for future structural and biophysical studies. This region in isolation is predicted to be intrinsically unstructured and is poorly conserved across species, but contains four highly conserved linear motifs arranged in a constant order. We propose that these are involved in interactions with importins (NLS), kinases (Ser patch) and maybe transcription factors in promoter complexes of auxin-sensitive genes implicated in floral organ morphogenesis.

## Methods

### Amplification and sub-cloning of the ETT gene

The *ETT* coding sequence was amplified by PCR from *Arabidopsis thaliana* Col-0 inflorescence cDNA with primers ETTfor (5′-ATGCA TGCGG CGCGC CTAGA TGGGT GGTTT AATCG ATCTG AACGT GATG-3′) and ETT-FLrev (5′-ATGCA TGCAT CCATT GAGAG CAATG TCTAG CAACA TGTCT CTCAT TGC-3′), ETT-MRMD-rev (5′-ATGCA TGCAT GCATT CATGT CTCTC ATTGC ATAGA TGTCC CTTCC TTG-3′) and ETT-SQGRrev (5′-ATGCA TGCAT GCATT CCTTC CTTGC GAATG ATGAT GATTG CTTTG-3′) to obtain full-length ETT (ending 608) and the two ETT truncated variants ETT-MRMD (ending 602) and ETT-SQGR (ending 594) respectively. Internal *Nsi*I sites were silenced by site-directed mutagenesis. The fragments were cloned in the pGEM-T-easy vector (Promega) and sequenced. Subsequently, fragments were excited from the pGEM-T vectors by digestion with *Asc*I and *Nsi*I and inserted in the pESPRIT002 vector^11^, designed for the 5′ truncation of the gene, and resulting in inserts fused to an N-terminal TEV-cleavable hexahistidine tag (MGHHHHHHDYDIPTTENLYFQG) and a C-terminal biotin acceptor peptide (SNNGSGGGLNDIFEAQKIEWHE).

### ESPRIT library screening for soluble domains of ETT

Full methods and materials for ESPRIT have been detailed recently^11^. Briefly, 5′ deletion libraries of the ETT gene were constructed on an equimolar pool of the three plasmids above using exonuclease III and mung bean nuclease and recovered into *E. coli* MACH1 (Thermo Fisher Scientific). The linear distribution of insert sizes was confirmed by colony PCR with insert-flanking primers. Plasmid DNA was purified from medium and large insert sub-libraries and used to transform *E. coli* BL21 AI RIL for expression screening. Sub-libraries were titrated on agar trays and 15,975 colonies (8550 for the medium sub-library and 7425 for the large sub-library) were picked robotically into 384-well plates, corresponding to a three-fold oversample of each clone, then arrayed onto nitrocellulose membranes over LB agar with antibiotics. Once visible colonies were apparent, membranes were transferred to LB agar containing antibiotics, arabinose and biotin for protein expression for 4 h at 30 °C. Membrane-supported colonies were lysed using NaOH and membranes neutralised and blocked with Superblock (Thermo Fisher Scientific). Membranes were hybridised with fluorescent probes: streptavidin Alexa 488 to detect *in vivo* biotinylation of the C-terminal biotin acceptor peptide on the target proteins, and antihexahistidine mouse monoclonal followed by Alexa532 labelled rabbit anti-mouse secondary antibody to detect the N-terminal hexahistidine tags. Quantitative analysis of fluorescence intensities of both signals per colony resulted in 95 putatively soluble clones re-arrayed into a destination plate. These were grown at 4 mL scale and hexahistidine-tagged proteins purified in a 96-well plate format with His6-MBP-BAP as a positive control. Eluted proteins were analysed by fluorescent streptavidin blot with fluorimagery, and InstantBlue (Merck) stained SDS-PAGE. The plasmid inserts from clones yielding visible purified proteins were DNA sequenced.

### Expression and purification of selected ETT clones

Clones were grown in 1 L LB medium (kanamycin 50 μg/mL and chloramphenicol 30 μg/mL) at 37 °C until OD_600_ 0.8. The temperature was reduced to 18 °C, and cultures were induced with 0.2% w/v L-arabinose overnight. Cells were harvested by centrifugation (11,000 g for 20 min at 4 °C) and the pellet frozen at −70 °C. Five grams of wet cell pellet were lysed by sonication in lysis buffer (8 M aqueous urea, 1 mM tris (2-carboxyethyl)phosphine (TCEP)), then centrifuged at 20,000 g, 40 min, 4 °C. Ni^2+^-NTA agarose resin (1.5 mL of 50% slurry) was added to the supernatant and incubated for 30 min at room temperature, then loaded on a gravity column. Five 50 mL wash steps were performed with buffers containing decreasing urea concentrations (8, 4, 2 and 1 M), 20 mM imidazole and 1 mM TCEP. A final wash was performed in equilibration buffer (50 mM Na phosphate pH 7, 50 mM NaCl, 20 mM imidazole, 1 mM TCEP), then purified proteins eluted in 1 mL fractions with elution buffer (50 mM Na phosphate pH 7, 50 mM NaCl, 300 mM imidazole, 1 mM TCEP). Total, soluble and unbound lysate samples, plus 10 elution fractions were analysed by 15% SDS-PAGE with staining by InstantBlue. For CD and thermal stability analyses, 1 mL of fraction 10 was dialysed twice against 1 L of Tris 5 mM pH 8.5 1 mM TCEP.

### Characterisation of purified ETT C-terminal domain

LC ESI-TOF analysis was performed by the IBS-ISBG mass spectrometry platform on an Agilent Technologies 6210 spectrometer coupled to a capillary HPLC system (1100 series). The far-UV CD spectrum (from 190 nm to 260 nm) was collected on a JASCO J-810 CD spectrophotometer. The ETT 388_594 sample at 6.5 µM in Tris 5 mM pH 8.5 1 mM TCEP was scanned ten times at 20 °C in a 1 mm path-length cuvette. After subtraction of baseline buffer values, the CD signal, expressed in mdeg, was converted to mean molar residue ellipticity (expressed in deg.cm2.dmol-1). Data were plotted using Prism (GraphPad Software, San Diego). The thermal stability analysis involved heating ETT 388_594 in the same buffer used for CD for 20, 40 and 60 min, followed by centrifugation at 13,000 g, 10 min. Supernatants were loaded on SDS-PAGE with an unheated centrifuged reference sample, electrophoresed and analysed after staining with InstantBlue.

### Yeast two-hybrid (Y2H) assay

The Y2H assays were performed at 28 °C in the yeast strain AH109 (Clontech) using the co-transformation technique^34^. Coding sequences were cloned into the Gateway vector GAL4 system (pGADT7 and pGBKT7; Clontech) via pDONR207 (Life Technologies). Strength of interaction was tested on selective yeast synthetic dropout medium (YSD) lacking Leu (L), Trp (W), Adenine (A), and His (H). IAA was dissolved in ethanol and added at the desired concentration directly to the cooling media.

### Plant Materials and Growth Conditions

Arabidopsis plants were grown on soil in long-day conditions (16 h light/8 h dark). T-DNA insertional mutants^23^ were obtained from the Nottingham Arabidopsis Stock Centre. *Brassica rapa* plants were grown in soil in 1 litre pots under long-day conditions (16 h light/8 h dark). Mutant lines *ett-3*, *arf3-1*, *arf3-2*, *Bra.A.ett.a.Stop220* have been described previously^24,35,36^. *Bra.A.ett.a.Stop220* and *Bra.A.ett.a.Gln480Stop* mutants can be found at http://revgenuk.jic.ac.uk/, codes JI32298-B and JI33329-B respectively.

### Scanning electron microscopy

Whole gynoecia and flowers were fixed 16 h at 25 °C in 3.7% formaldehyde, 5% glacial acetic acid, and 50% ethanol. After a complete dehydration through an ethanol series until 100%, gynoecia were critical point dried. Samples were dissected, mounted on metal stubs and coated with gold and examined under Zeiss Supra 55VP field emission scanning electron microscope using an acceleration voltage of 3 kV.

### Error-prone screening of ETT ES domain

Error-prone PCR mix was composed of G2-polymerase buffer (Promega) and G2-polymerase, 5 mM MgCl_2_, 0.15 mM MnCl_2_, unbalanced dNTP mix (2.5 mM dATP, 2.5 mM dGTP, 10 mM dCTP, 10 mM dTTP). The pool of PCR fragments was incubated with the pGADT7-rec plasmid (Clontech) following the co-transformation protocol described in the “Matchmaker Library Construction & Screening User Manual (Clontech)”. Cells were plated on YSD selective media lacking Trp (W), Leu (L), Ade (A) and His (H) and supplemented with 100 μM IAA. Plasmids were recovered from those colonies able to grow in presence of IAA and sequenced.

## Electronic supplementary material

Supplementary Information
