# Supplementary material for: Auxin sensing is a property of an unstructured domain in the Auxin Response Factor ETTIN of Arabidopsis thaliana
Source: Sci Rep. 2018 Sep 10;8:13563. doi: 10.1038/s41598-018-31634-9 (PMC6131142; doi:10.1038/s41598-018-31634-9)
Supplement: Supplementary file 1 — Supplementary Information [file 41598_2018_31634_MOESM1_ESM.pdf]

Supplementary Information

Auxin sensing is a property of an unstructured domain in the Auxin Response Factor ETTIN of *Arabidopsis thaliana*

Sara Simonini, Philippe J. Mas, Caroline M. V. S. Mas, Lars Østergaard and Darren J. Hart

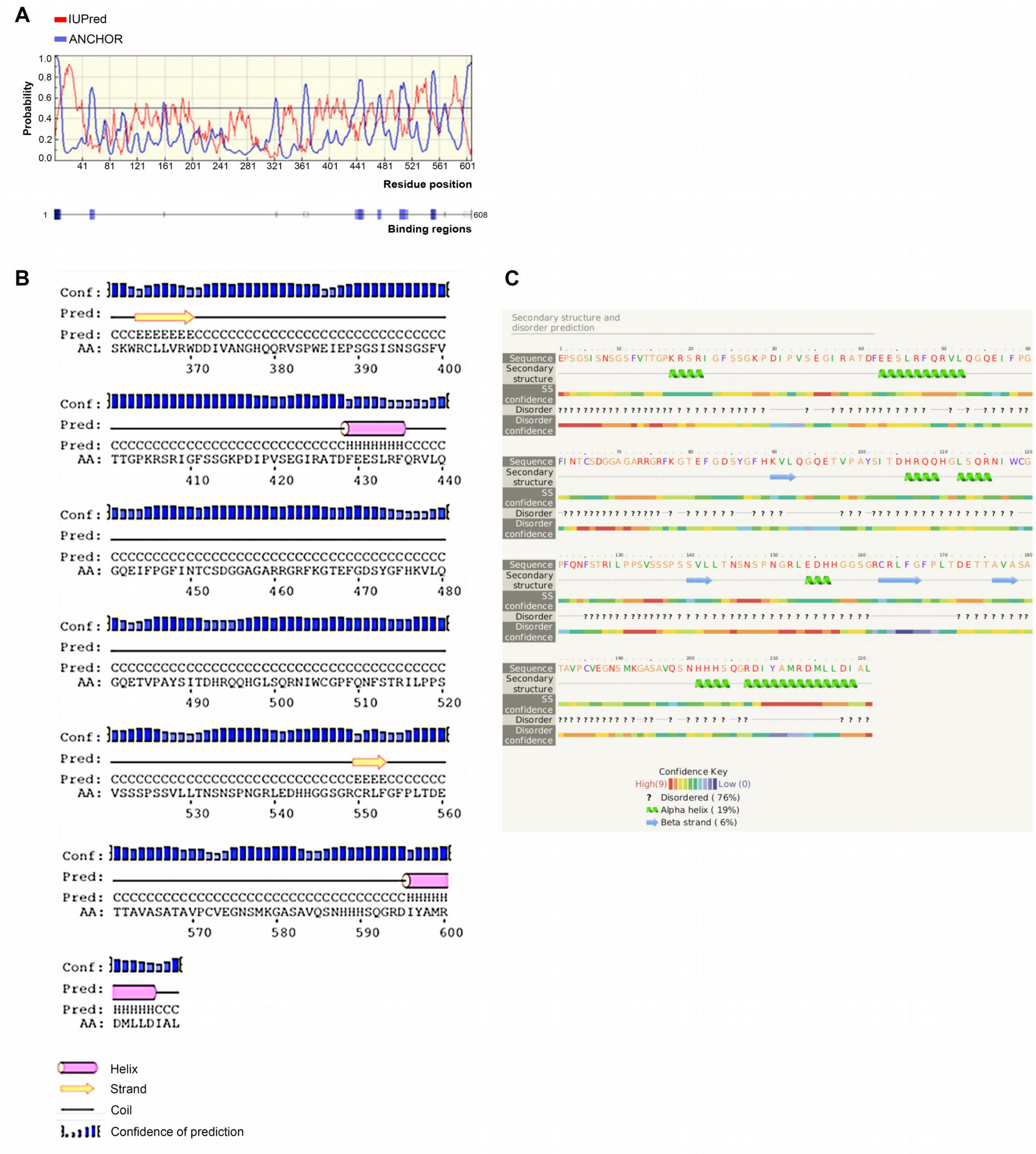

Supplementary Figure 1.

Structure prediction by (A) IUPred indicating predicted disorder in red and potential interaction sites in blue; (B) PsiPRED indicating secondary structure; and (C) Phyre2 indicating predicted disorder and secondary structure.

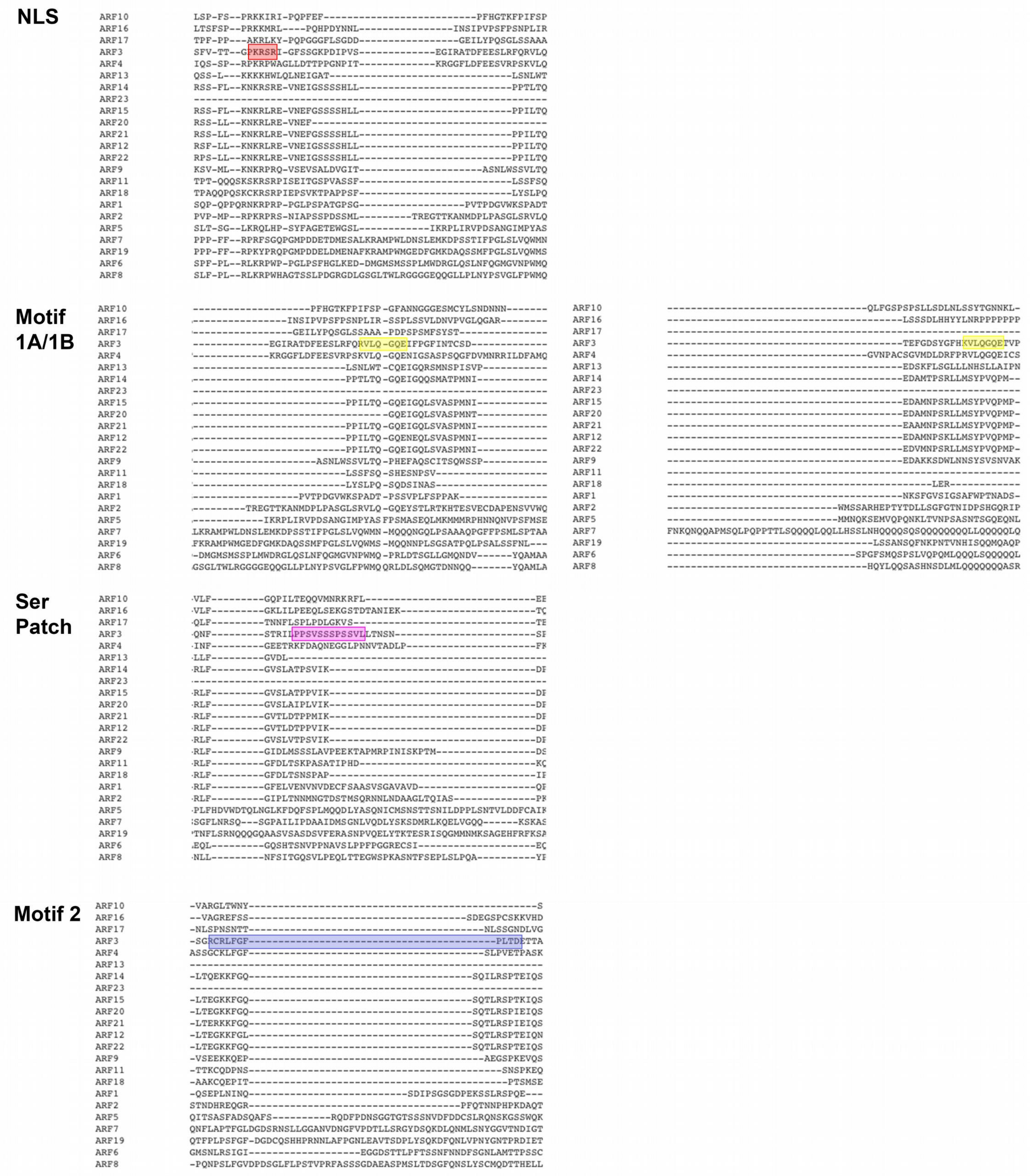

Supplementary Figure 2.

Amino acid alignment of the 23 ARFs of *A. thaliana*

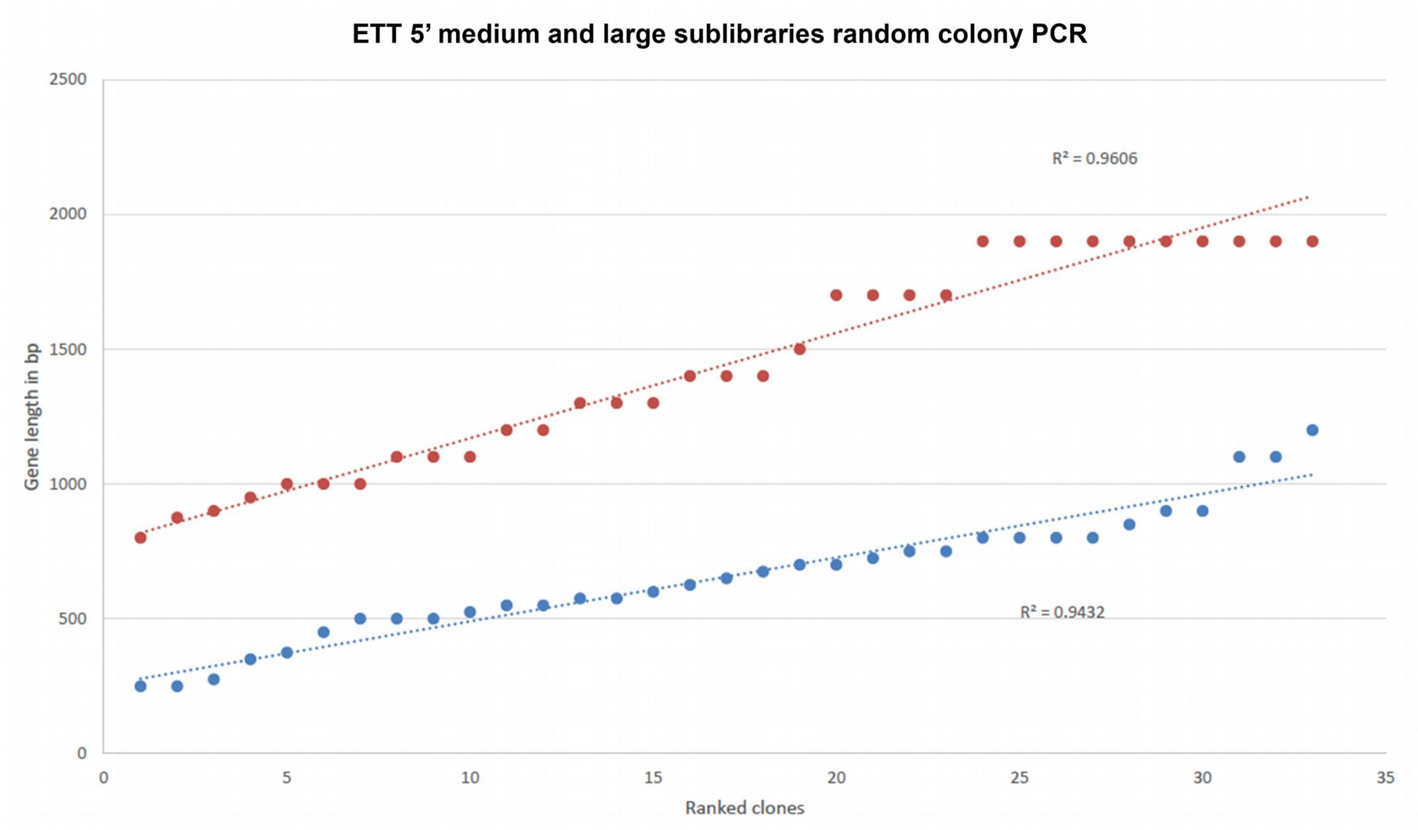

Supplementary Figure 3.

Ranked plot of *ETT* gene insert lengths (bp) by colony PCR of randomly selected clones from medium and large insert sublibraries showing even distribution of construct sizes in both. Correlation coefficients (R^2) are shown for both sublibraries.

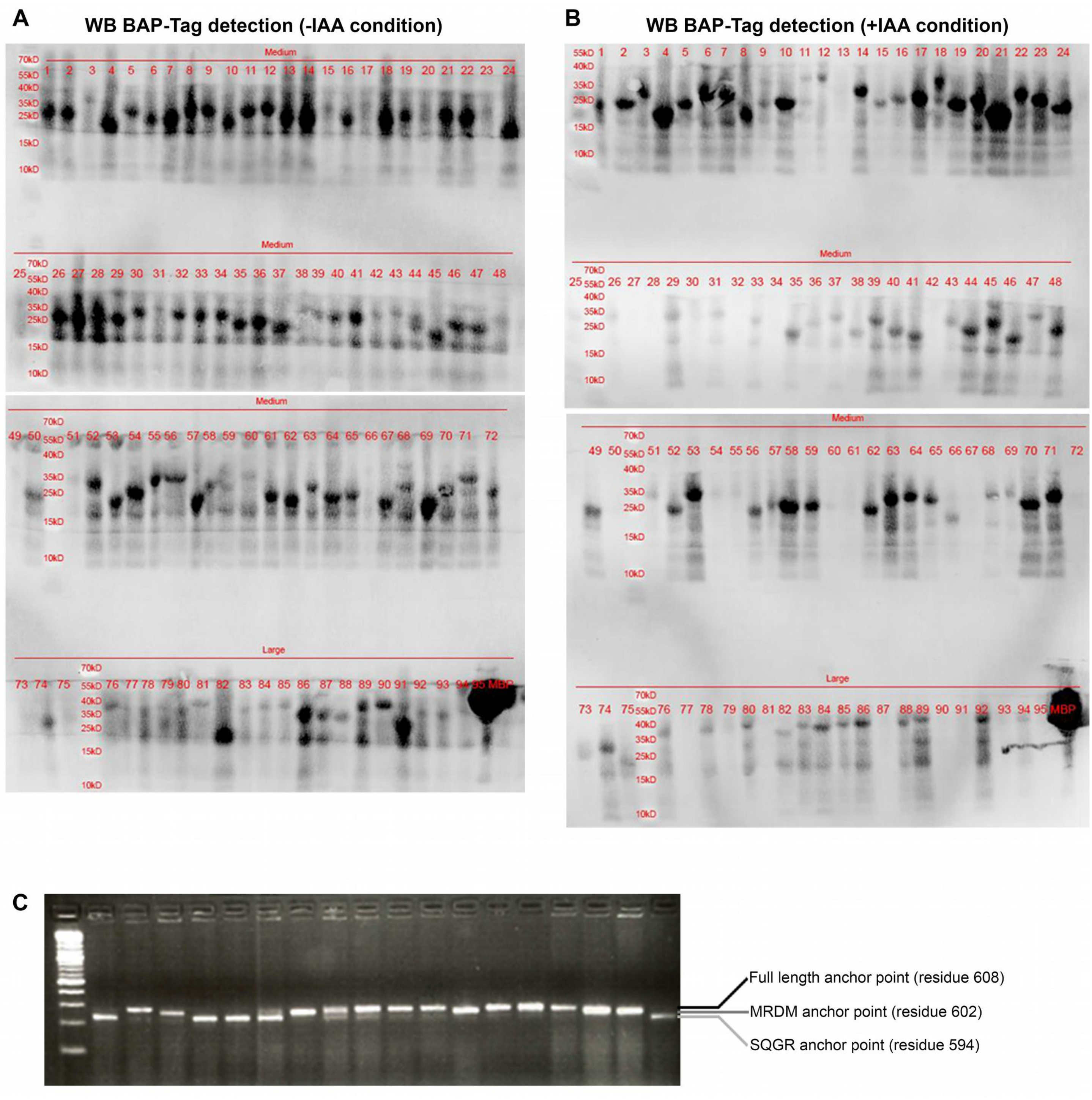

Supplementary Figure 4.

(A-B) Fluorescent streptavidin blot against C-terminal biotin acceptor peptide following Ni^{2+}-NTA purifications from 4 mL small-scale cultures of the 95 selected clones from (A) Minus (-)IAA and (B) Plus (+)IAA conditions plus 6xHis-MBP-BAP control (lane 96). The first three gels show clones from the medium sub-library (DNA fragments from 250 to 1200 bp); the last gel from the large sub-library (DNA fragments from 800 to 1900 bp). Lanes correspond to constructs shown in Fig. 3 as follows: 442-594, gel +IAA, lane 4; 439-594, gel -IAA, lane 4; 416-594, gel -IAA, lane 54; 415-594, gel -IAA, lane 18; 413-594, gel -IAA, lane 27; 397-594, gel -IAA, lane 29; 396-594, gel -IAA, lane 26; 391-594, gel -IAA, lane 19; 388-594, gel -IAA, lane 8; 380-594, gel -IAA, lane 33; 373-594, gel -IAA, lane 9; 340-594, gel -IAA, lane 86.

(C) Agarose gel showing insert sizes by colony PCR on randomly selected clones from the library prior to screening confirming that all the three fixed 3' ends were present.

A

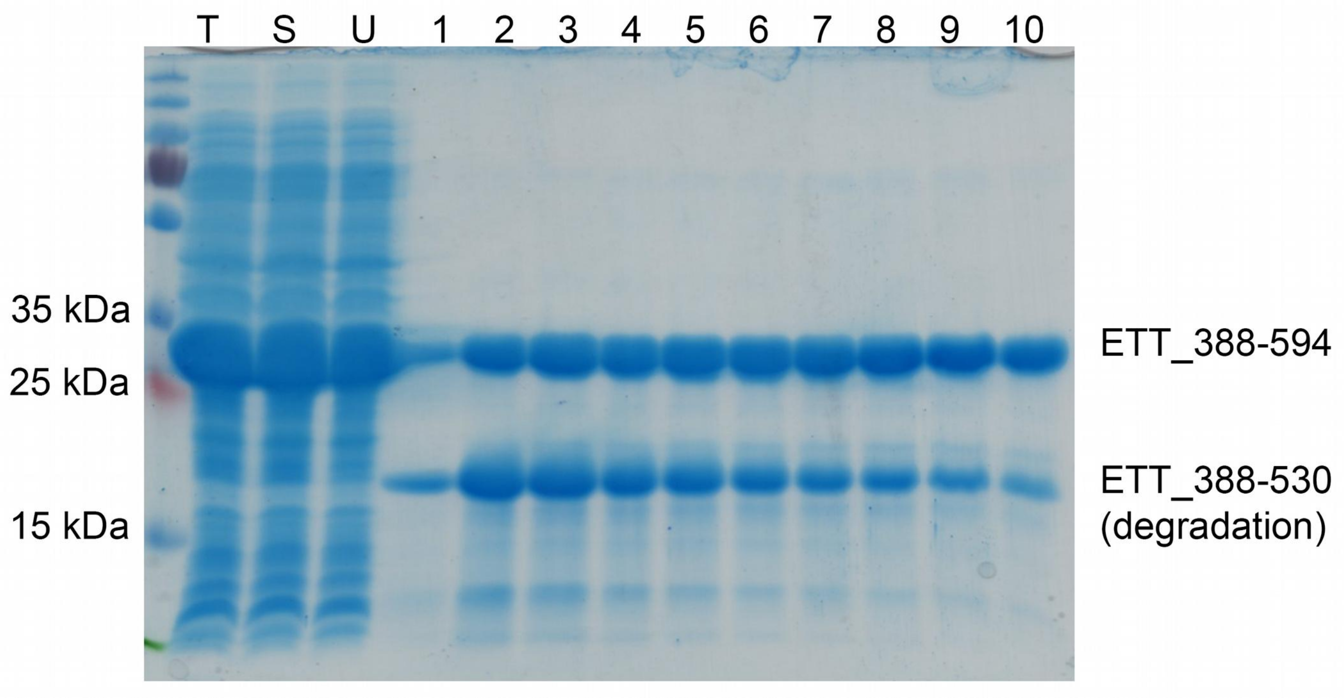

B

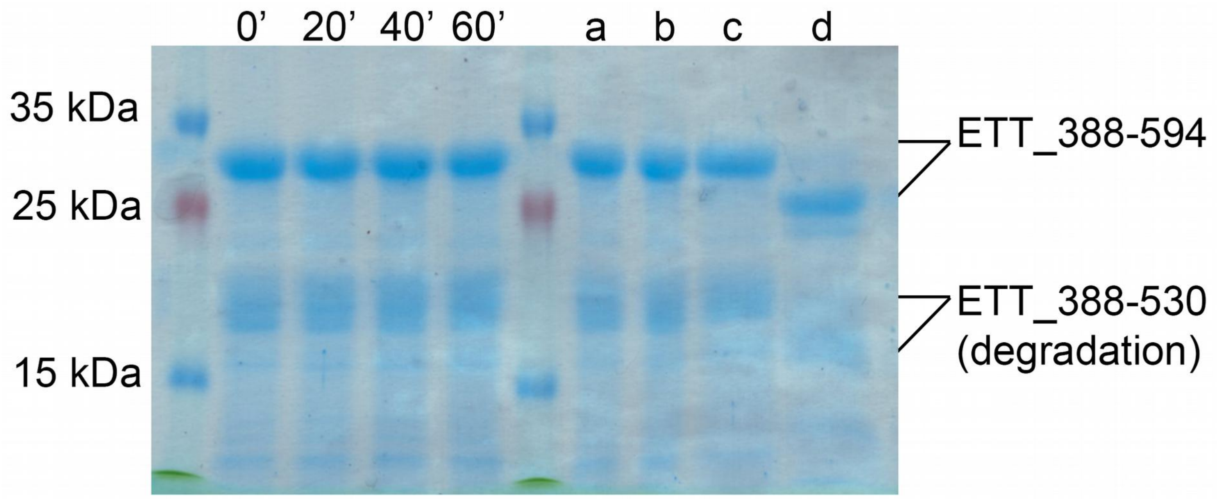

Supplementary Figure 5.

(A) SDS-PAGE analysis of Ni^{2+}-NTA purified ETT_388-594 with hexahistidine and biotin acceptor peptide tags from 2 L of bacterial culture. Elution fractions comprised a strong major species and a minor component of lower molecular weight. T; Total fraction, S: soluble fraction; U: unbound fraction during resin binding step; 1-10: 1 mL elutions. Fraction 10 was used for CD analysis and thermal stability assays.

(B) Left side of gel: Thermal stability assay of purified tagged ETT_388-594 (fraction 10). Protein was heated at 95 °C for 0, 20, 40 and 60 minutes and the soluble fraction analysed after centrifugation. No change in solubility was observed. Right side of gel: lane a: ETT_388-594 after 1 week at RT; lane b: sample from lane a after centrifugation (no precipitation observed); lane c: overnight mock digestion without TEV protease; d: overnight digestion with TEV protease.

GGAAGAGGGTGATGTTGATACAGATGG	ETT ES S523T rev
CCATCTGTATCATCAACACCCTCTTCCG	ETT ES S524T fw
CGGAAGAGGGTGTTGATGATACAGATGG	ETT ES S524T rev
ATCATCACCCACTTCCGTCTTGC	ETT ES S526T fw
GCAAGACGGAAGTGGGTGATGAT	ETT ES S526T rev
CACCCTCTACCGTCTTGCTTACCA	ETT ES S527T fw
TGGTAAGCAAGACGGTAGAGGGTG	ETT ES S527T rev
TCTAGAGGCCGAGGCGGCCGACATGCTAGAGAGCAATGTCTAGCAAC	REV on ETT for pGADT7 recombination
AAGCAGTGGTATCAACGCAGAGTGGCCATTATGGCCATGATCTCCAATTCAGGCAGCTTCG	FW ETT-ES domain for error-prone analyses pGADT7 rec
GGGGACAAGTTTGTACAAAAAAGCAGGCTTCATGGAACCATCTGGTTCCATCTCCAATTC	ETT ESPRIT clone fw gw
GGGGACCACTTTGTACAAGAAAGCTGGGTGCTACCTTCCTTGCGAATGATGATGATTGC	ETT ESPRIT clone rev gw (ends in SQGR)

Supplementary Figure 6.

Table of primers used in this study. Sequences are shown 5' to 3'.
